# Polarized photoreflectance and photoluminescence spectroscopy of InGaAs/GaAs quantum rods grown with As_2_ and As_4_ sources

**DOI:** 10.1186/1556-276X-7-609

**Published:** 2012-11-05

**Authors:** Ramūnas Nedzinskas, Bronislovas Čechavičius, Julius Kavaliauskas, Vytautas Karpus, Gintaras Valušis, Lianhe Li, Suraj P Khanna, Edmund H Linfield

**Affiliations:** 1 Optoelectronics Department, Semiconductor Physics Institute, Center for Physical Sciences and Technology, A. Goštauto 11, Vilnius, 01108, Lithuania; 2 School of Electronic and Electrical Engineering, University of Leeds, Leeds, LS2 9JT, UK; 3 CSIR-National Physical Laboratory, 110012, Dr. K. S. Krishnan Marg New Delhi, India

**Keywords:** InGaAs quantum rods, Optical transitions, Electronic structure, Photoreflectance, Photoluminescence, Optical anisotropy, 78.55.Cr; 78.67.Hc; 78.67.Qa

## Abstract

We report photoreflectance (PR) and photoluminescence (PL) investigations of the electronic and polarization properties of different aspect ratio (height/diameter) InGaAs quantum rods (QRs) embedded in InGaAs quantum wells (QWs). These nanostructures were grown by molecular beam epitaxy using As_2_or As_4_sources. The impact of the As source on the spectral and polarization features of the QR- and QW-related interband transitions was investigated and explained in terms of the carrier confinement effects caused by variation of composition contrast between the QR material and the surrounding well. Polarized PR and PL measurements reveal that the polarization has a preferential direction along the $\left[1\bar{1}0\right]$ crystal axis with a large optical anisotropy of about 60% in the (001) plane for high aspect ratio (4.1:1) InGaAs QRs. As a result, in PL spectra, the transverse magnetic mode dominated $\left(1\bar{1}0\right)$-cleaved surfaces (TM_[001]_>TE_[110]_), whereas the transverse electric mode prevailed for (110)-cleaved surfaces (${\text{TM}}_{\left[001\right]}<{\text{TE}}_{\left[1\bar{1}0\right]}$). This strong optical anisotropy in the (001) plane is interpreted in terms of the hole wavefunction orientation along the $\left[1\bar{1}0\right]$ direction for high aspect ratio QRs.

## Background

Self-assembled semiconductor quantum dots (QDs) formed by molecular beam epitaxy (MBE) are the foremost candidates for numerous applications in optoelectronics (see e.g., [1]). Modifying the polarization-dependent optical gain function is, however, important for optoelectronic engineering. For this reason, columnar QDs or quantum rods (QRs) have been grown by MBE by depositing a short period InAs/GaAs superlattice (SL) on top of a seed QD layer [2]. The active region of the resulting nanostructures thus comprises InGaAs QRs immersed in a 2-D InGaAs layer.

In addition to being able to engineer the transverse electric (TE) and transverse magnetic (TM) optical mode couplings, which is very beneficial for semiconductor optical amplifiers (SOAs), QRs have a large intrinsic dipole moment, which opens up opportunities for their application in quantum memories and nonlinear electro-optic devices [3]. However, structural (TEM) analysis of such elongated nanostructures gives clear evidence of in-plane shape anisotropy [2]. This is confirmed by very recent theoretical and experimental optical studies which demonstrate the presence of optical anisotropy in multilayer InAs/GaAs QD stacks [4]. Material composition gradients, asymmetric strain distributions, piezoelectrical effects, and even bending of the stacking direction during growth have all been used to explain the large observed optical anisotropies. Yet, it is clear that further work is required to elucidate the underlying mechanisms.

In this report, the optical properties and electronic structure of QRs are investigated using polarization-resolved photoreflectance (PR) and photoluminescence (PL) spectroscopies [5], techniques which have already proved to be very productive for studying QDs [6] and QRs [7,8]. The effects of the QR height (number of SL periods deposited) along with the As source (As_2_or As_4_) used in the MBE growth on the polarization properties and electronic structure of InGaAs QRs are mostly considered. Particular emphasis is placed on spectral features associated with interband optical transitions occurring in the InGaAs QRs and the surrounding InGaAs quantum well (QW) regions for As_2_/As_4_-grown nanorod structures.

Whilst in previous studies, the polarization response of QDs and QRs was investigated for a single undefined TE mode [9], our polarization-resolved PR and PL measurements revealed a unique property of InGaAs QRs; the TE response is anisotropic in the (001)-plane. Therefore, the polarization response of QRs cannot be fully characterized by a single TE mode.

## Methods

### Samples and experimental techniques

Two sets of InGaAs QR samples were grown by MBE under the same growth conditions but with different As sources (As_2_ and As_4_) on (001)-oriented GaAs substrates. After a GaAs buffer layer, 200-nm thick Al_0.2_Ga_0.8_As and 100-nm thick GaAs layers were grown. The self-assembled QRs were formed by first depositing 1.8-monolayers (MLs) of InAs, which relaxed under Stranski-Krastanow conditions into a QD seed layer. A short period SL of alternating GaAs (3 ML) and InAs (0.64 ML) epilayers, followed by a 100-nm thick GaAs capping layer, then completed the structure. To investigate the effects of the As source and QR morphology on the optical properties of the QR structures, two sets of QR samples were grown with SL periods *N*=10, 20, and 35 which are designated as QR10, QR20, and QR35, respectively. It should be noted that control of the height of the QRs can be achieved both by adjusting the thicknesses of the InAs and GaAs epilayers, as well as by changing the number of periods (*N*) in the SL [10]. The estimated heights of the QRs were 20 nm (QR10), 32 nm (QR20), and 41 nm (QR35).

Room temperature spectroscopy of InGaAs QR structures was performed using PR and PL techniques. We also used linearly polarized PR (PPR) and PL (PPL) to reveal both the in-plane optical anisotropy, and the cleaved facet polarization properties of QRs. In PPR experiments, the incident angle of the light was less than 10 ^∘^. He-Ne (632.8 nm) and diode-pumped solid-state (473 and 532 nm) lasers were used as the modulation (PR) and excitation (PL) sources. PR and PL signals were recorded with a thermoelectrically cooled InGaAs or Si detector using conventional lock-in techniques.

## Results and discussion

**Figure 1:**
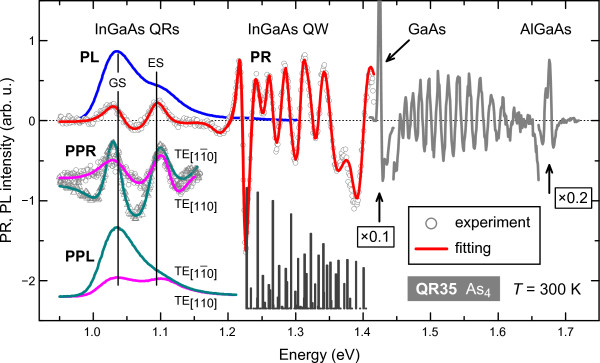
**Room temperature PR and PL spectra of the As_4_-grown sample QR35.** PPR and PPL spectra show a significant in-(001)-plane polarization anisotropy in the InGaAs QR-related interband transitions for the $\left[1\bar{1}0\right]$ (green curves) and [110] (pink curves) polarizations. Vertical bars indicate numerically evaluated energies and relative strengths of the interband transitions in the InGaAs QW.

Room temperature PR and PL spectra for the As_4_-grown sample QR35 are shown in Figure 1. The PR spectrum splits into four principal sets of optical features, which correspond to specific optical transitions in the QR structures [7]. An optical feature at 1.67 eV results from the bandgap transition in the Al_0.2_Ga_0.8_Aslayer. Pronounced oscillations in the spectral range of 1.42 to 1.67 eV can be attributed to the above GaAs barrier states. Optical features between 1.2 to 1.4 eV correspond to the optical transitions occurring in the InGaAs QW that surrounds the QRs. Finally, optical PR features in the 1 to 1.2 eV region lie below the broad PL curve and, therefore, correspond to the ground- (GS) and excited-state (ES) transitions in the QRs.

To determine the transition energies and broadening parameters, the recorded PR features (symbols in Figure 1) were fitted to the first derivative of a Lorentzian-type function [5,11,12], while transition intensities were defined from the PR modulus spectra. Spectroscopic data were interpreted by numerical calculations within the envelope function approximation using *nextnano*^3^software [13] (dr. Stefan Birner, Poing, Germany).

The vertical bars in Figure 1 indicate the calculated relative strengths (overlap integrals) of the optical transitions in the InGaAs QW under zero electric field (flat band) conditions.

The characteristic rhombus shape of the QRs is clearly visible in (001)-plane-view TEM images [2], suggesting that optical anisotropy needs to be considered. We, thus, analyzed the linear polarization properties for light propagating in the growth direction. As can be seen from Figure 1, linearly PPR and PPL spectra revealed a significant polarization anisotropy in the (001) plane in the spectral region corresponding to InGaAs QR-related interband transitions; these will be considered in detail hereinafter.

### Carrier confinement in InGaAs QRs

**Figure 2:**
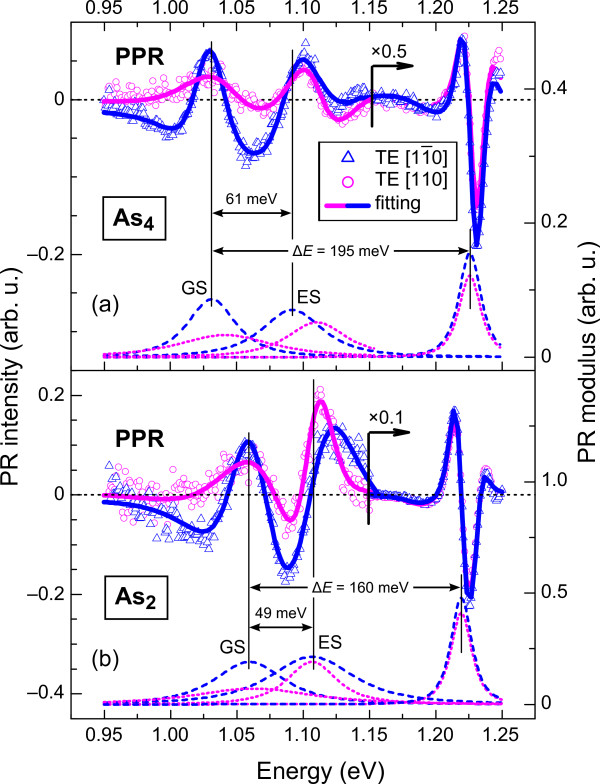
**Room temperature PPR spectra at two perpendicular polarization angles (symbols).** (**a**) As_4_-grown and (**b**) As_2_-grown QR35 samples. GS and ES denote optical transition energies in InGaAs QRs, involving ground and excited states, respectively. The modulus of individual PPR resonances are represented by dashed and dotted lines for the electric vector ***E***being parallel to the $\left[1\bar{1}0\right]$ and [110] crystallographic axes, respectively.

We initially studied the effect of the As (As_2_and As_4_) source used during MBE growth on the optical properties of QRs. A comparison of room temperature PPR spectra at two perpendicular polarization angles for As_4_- and As_2_-grown QR35 samples (Figure 2) illustrates some notable characteristics. In particular, the low-energy QR-related features in the experimental spectra are red shifted for the As_4_-grown structure compared to the As_2_-grown samples. In contrast, there is a blue shift in the QW-related features. Moreover, the intensity of the PL (not shown) and PR signal is significantly enhanced when an As_4_source is used.

The relative red shift of QR-like and blue shift of QW-like optical transitions for the As_4_-grown QR sample were analyzed in terms of the carrier confinement, defined as the energy spacing *ΔE* between the lowest QW-related transition and the QR ground-state transition. By following the corresponding peak energies of the PPR modulus (dashed and dotted curves in Figure 2), one can ascertain that the use of an As_4_ flux results in better carrier confinement compared to the use of As_2_; the energy spacings *ΔE*are 195 and 160 meV, respectively. Such an increase in carrier confinement for As_4_-grown QRs is also evident from the significantly larger energy spacing between QR ground- (GS) and excited- (ES) states for the As_4_-grown sample (61 meV) compared to the As_2_-grown one (49 meV). The increased energy level spacing in the As_4_-grown QR structures may be attributed to the electron wavefunctions being more tightly confined, as discussed in [14]. This could be important for improving the recombination efficiency and PL intensity in QR devices, such as SOAs. These results suggest that there is better carrier confinement in As_4_-grown QRs because of an increased indium composition contrast between the InGaAs QRs and the surrounding InGaAs QW layer [7].

### Polarization properties of InGaAs QRs and QWs

Optical anisotropy in the (001) plane of InGaAs nanorods was explored by PPR and PPL using two linear light polarizations, along the $\left[1\bar{1}0\right]$ and [110] crystal axes. Room temperature PPR spectra at these two perpendicular polarization angles (Figure 2) revealed significantly different PPR signal intensities for QR-related optical features both for As_4_- and As_2_-grown samples. This optical anisotropy was confirmed by PPL measurements (Figure 1). However, for the QW-related transitions, a negligible polarization dependance was observed.

To gain a deeper insight into the effect of the QR aspect ratio (height/diameter) on the optical anisotropy of InGaAs QRs, we systematically analyzed the PPR and PPL responses as a function of SL period number *N*. The polarized PR and PL spectra (Figures 1 and 2) were evaluated by a quantitative measure, the degree of polarization (DOP):

(1) $${\mathrm{DOP}}_{\left(001\right)}=\frac{I_{\left[1\bar{1}0\right]}-I_{\left[110\right]}}{I_{\left[1\bar{1}0\right]}+I_{\left[110\right]}}.$$

Here, $I_{\left[1\bar{1}0\right]}$ and *I*_[110]_ denote signal intensities for $E∥[1\bar{1}0]$ and ***E***∥[110]light polarizations (two perpendicular polarizer positions), or alternatively for two transverse electric modes, ${\mathrm{TE}}_{\left[1\bar{1}0\right]}$ and TE_[110]_, respectively.

**Figure 3:**
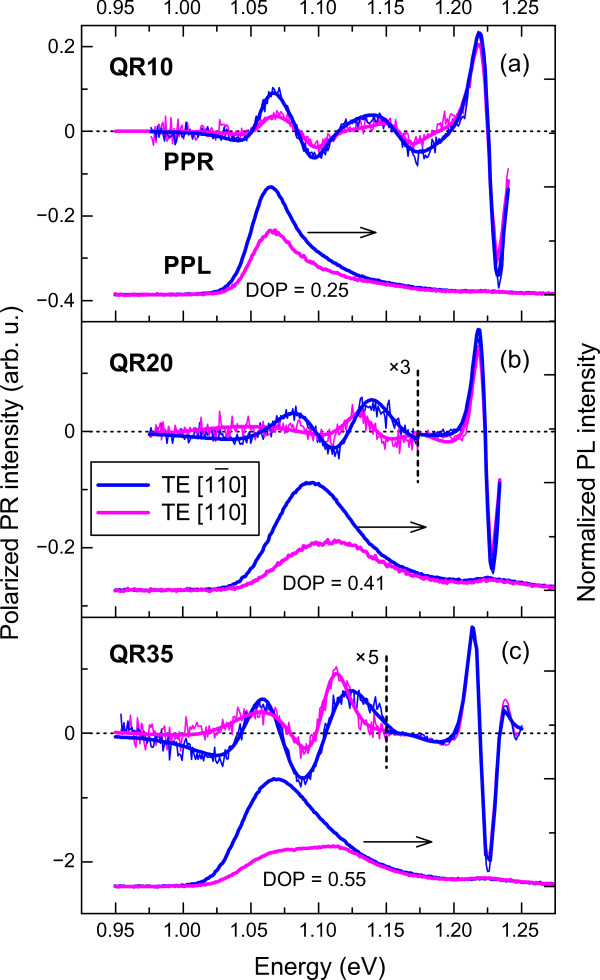
**Room temperature PPR and PPL spectra in the (001) plane for the As_2_-grown QR samples.** (**a**) QR10, (**b**) QR20, and (**c**) QR35. PPL optical anisotropy of the GS interband transitions in the QRs is indicated by the DOP values.

Figure 3 shows room temperature PPR and PPL spectra at two perpendicular polarization angles in the (001) plane for the As_2_-grown QR samples: (a) QR10, (b) QR20, and (c) QR35. From the PPL data, it was found that the in-plane degree of polarization, DO*P*_(001)_, increases almost linearly with the SL period number *N*. In particular, for small aspect ratio (2.0:1) QRs (QR10), the degree of polarization is small, *DOP*_(001)_≈25*%*, and comparable to the DOP values for conventional self-assembled QDs. Increasing the SL period to *N*=20 (aspect ratio: 3.2:1) results in an increase of DOP value to ≈41*%*. Finally, for high aspect ratio QRs of 4.1:1 (QR35), the in-(001)-plane DOP reaches the value of ≈55*%*, close to the value (DOP_(001)_≈60*%*) estimated for the As_4_-grown QR35 structure.

The polarization anisotropy, estimated from the analysis of PPR modulus spectra, shows similar increase with SL period number *N*, however to a smaller extent. In particular, for the samples QR10, QR20, and QR35, we estimated DOP values of 37%, 43%, and 46%, respectively.

The significantly different DOP values obtained from PPR and PPL spectra (Figure 3) may be related to the fact that PR is an absorption-based spectroscopic method, which probes the maximum in the density of states, whilst PL probes the states of lowest energy. Alternatively, the different DOP values may be due to interference effects in the PR signal [15].

In general, for low-dimensional structures, the PR signal intensity is associated with the quantum-confined Stark effect (QCSE), the squared overlap integral of the electron and heavy-hole wavefunctions, |*M*_cv_|^2^, modulation efficiency, and the built-in electric field [16]. In QRs, the lowest state electron wavefunction spans the whole length of the QR, whereas the hole wavefunctions of the two lowest states are well-localized at the top and the bottom of the QR. This confinement of holes is mainly driven by the large heavy-hole effective mass and strain-related modification of confinement potential in the growth direction [17]. The recorded PR signal of ground-state transition in a QR is thus a superposition of two signals involving two well-confined hole states. Due to the difference in optical paths (the probed areas are spatially separated over the QR height), the contributed signals may arrive with a different phase and thus interfere constructively or destructively.

It is tentatively suggested that under influence of an electric field (the QCSE), with localization of the lowest heavy-hole states at the top and the bottom of the QR, the recorded PR intensity is modified due to the interference of the two spatially separated signals. This can be further explored using different modulation sources for the PR experiments.

The PPR spectra in Figure 3 also show a decrease of QR-related transition intensities relative to the lowest energy peak in the QW as the number of periods, *N*, in the SL increases. This behavior is attributed to a decrease in the overlap integral of the electron and heavy-hole wavefunctions with increase of QR height.

It should be noted that the strong anisotropy in the (001) plane cannot be explained purely in terms of the in-plane elongated shape of the QR along the $\left[1\bar{1}0\right]$ direction which is normally up to about 30%. Other possible effects leading to the polarization asymmetry are material composition gradients (fluctuations), asymmetric strain distributions, piezoelectricity, and in-plane modulation of the InGaAs content — all need to be considered equally. For example, material composition fluctuations involving valence band mixing cause up to 40% polarization anisotropy, even in highly symmetrical QDs [18]. However, the situation in QRs is even more complicated. Recent TEM observations by Mukai et al. [19,20] suggested that polarization features may be governed by problems in the growth process such as the bending of the stacking direction during the formation of columnar QDs with a high aspect ratio. However, a very recent optical anisotropy investigation of stacked InAs/GaAs QD structures [4] revealed that a large optical anisotropy could be ascribed to the hole wavefunction orientation along $\left[1\bar{1}0\right]$ axis, which suppresses the TE_[110]_mode.

Our experimental findings suggest that the PL polarization properties (intensity of TE versus TM mode) from cleaved facet surfaces should be different for the (110) and $\left(1\bar{1}0\right)$ facets. When considering the optical anisotropy from cleaved facet surfaces [$\left(1\bar{1}0\right)$ and (110) planes] of QR samples, the degree of polarization can be defined by

(2) $${\mathrm{DOP}}_{\left(\mathrm{hkl}\right)}=\frac{{\mathrm{TE}}_{⊥\text{-}z}-{\mathrm{TM}}_{∥\text{-}z}}{{\mathrm{TE}}_{⊥\text{-}z}+{\mathrm{TM}}_{∥\text{-}z}},$$

where *z* is the QR growth direction, and TE (TM) is the intensity of the transverse electric (magnetic) mode. In this case, the DOPis characterized by the Miller indices of the crystallographic facet plane.

**Figure 4:**
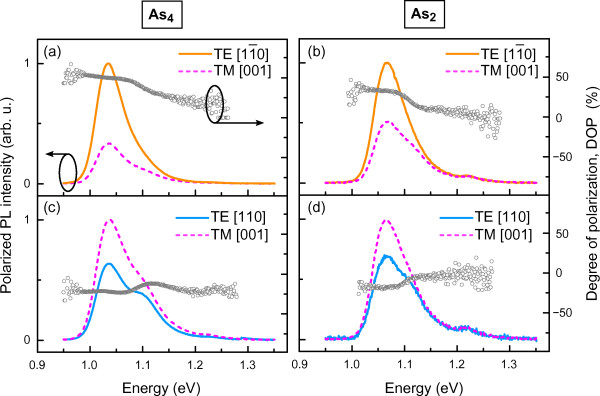
**Room temperature linearly polarized photoluminescence spectra split into TE and TM modes for QR35 samples.** The samples were grown using As_4_(**a**, **c**) and As_2_(**b**, **d**) sources. PL spectra are normalized to the most intense peak of the GS transitions in the InGaAs QRs. Circles indicate the optical anisotropy, represented by the degree of polarization, DOP.

Room temperature, linearly polarized PL spectra at two perpendicular polarization angles (Figure 4) show that for high aspect ratio (4.1:1) QRs (QR35), a flip of DOP sign occurs for both As_4_- and As_2_-grown InGaAs QR structures. As a result, the TM mode is dominant from the $\left(1\bar{1}0\right)$ surface (TM_[001]_>TE_[110]_), whilst from the (110) surface, the TE mode prevails (${\mathrm{TM}}_{\left[001\right]}<{\mathrm{TE}}_{\left[1\bar{1}0\right]}$). The DOP values for As_4_-grown QRs (Figure 4a,c) of DOP_(110)_= + 50*%* and ${\mathrm{DOP}}_{\left(1\bar{1}0\right)}=-23\%$ exceed the corresponding DOP values of DOP_(110)_= + 33*%*and ${\mathrm{DOP}}_{\left(1\bar{1}0\right)}=-18\%$ for As_2_-grown QRs (Figure 4b,d).

It should be noted that in lens-like QDs, optical transitions involving light-hole states are suppressed (by highly negative biaxial strain); therefore, the intensity of the TM mode becomes insignificant. As the QR height increases, biaxial strain reduces, simultaneously decreasing heavy- and light-hole sub-band splitting. Therefore, for high aspect ratio QRs, the light- and heavy-hole bands are almost degenerate and, thus, manifest themselves by TE and TM modes of comparable intensity. It is thus suggested that light- and heavy-hole sub-band mixing favors an increase of the TM_[001]_ mode. This is supported by calculated hydrostatic and biaxial strain profiles for InAs/GaAs QD stacks of different heights, as reported in ref. [4]. Finally, the evidence of sign reversal in the degree of polarization estimated from the PPL spectra fulfills the promise of QD shape engineering and shows a huge potential for QRs for potential applications, such as polarization insensitive SOAs.

## Conclusions

Polarized photoreflectance and photoluminescence spectroscopies were used to study the electronic structure and optical anisotropy of InGaAs QR structures. It was found that use of an As_4_ source, rather than an As_2_source, during MBE growth resulted in a better carrier confinement and overall performance of QR structures.

Spectroscopic investigations of InGaAs QRs demonstrated that the degree of polarization in the (001) plane reaches a high value of about 60% for InGaAs QRs with a large SL period number (*N*=35), owing to the hole wavefunction distribution along the $\left[1\bar{1}0\right]$ direction. As a result, the PL polarization properties (TE and TM modes) from cleaved facet surfaces are different for the (110) and $\left(1\bar{1}0\right)$ facets. The TM mode was dominant from the $\left(1\bar{1}0\right)$ surface (TM_[001]_>TE_[110]_), whilst from the (110) surface the TE mode prevailed (${\mathrm{TM}}_{\left[001\right]}<{\mathrm{TE}}_{\left[1\bar{1}0\right]}$). These spectroscopic findings provide fundamental knowledge of the polarization properties of QRs, which is essential for future engineering of a broad range of optoelectronic devices.

## Competing interests

The authors declare that they have no competing interests.

## Authors’ contributions

RN carried out some of the experimental measurements and drafted the manuscript. LHL, SPK, and EHL prepared the quantum rod samples using molecular beam epitaxy. BČ designed the experiment and performed the measurements. JK conceived the experimental set-up and provided possible explanations for the results. JK together with VK made simulations within envelope function approximation and participated in the discussions. GV supervised the project. All authors read and approved the final manuscript.

## Authors’ information

RN has recently obtained his Ph.D. degree in Physics at Vilnius University, Lithuania. RN is interested in semiconductor quantum dot-based structures in III-V material system. BČ has obtained his Ph.D. degree in Physics at Vilnius Gediminas Technical University, Lithuania in 2003. He is currently a research associate in Semiconductor Physics Institute. BČ takes interest in modulation spectroscopy experimental techniques and performs calculations using nextnano^3^ software. Dr. JK is a senior researcher in Semiconductor Physics Institute. His research interests are in the area of optical properties in low-dimensional systems. Dr. VK is a senior researcher in Semiconductor Physics Institute and a lecturer in Vilnius University. His research interests are in the area of simulations in low-dimensional systems and quasicrystals. Prof. GV is a principal investigator and head of the Semiconductor Physics Institute. His fields of scientific interests include low-dimensional systems, terahertz devices, superlattices, and Bloch oscillations. LHL received his Ph.D. degree in Microelectronics and Solid-State Electronics from the Institute of Semiconductors, Chinese Academy of Sciences, Beijing, China, in 2001. He now works with molecular beam epitaxy at School of Electronic and Electrical Engineering. SPK received his Ph.D. degree from the University of Leeds, UK in 2008. He is now a principal scientist at the National Physical Laboratory, India. His research interests involve developing semiconductor materials and devices for electronic, optoelectronic, and spintronic applications using III-V material system. Prof. EHL is the chair of Terahertz Electronics and director of research at the School of Electronic and Electrical Engineering. His field of interests include Condensed Matter Physics, Optics and Optoelectronics, and Electrical and Electronic Engineering.

## References

[B1] Bimberg D Grundman M Ledentsov NN Quantum Dot Heterostructures 1999 Chichester: Wiley

[B2] Li LH Patriarche G Rossetti M Fiore A Growth and characterization of InAs columnar quantum dots on GaAs substrate J Appl Phys 2007 102 033502 10.1063/1.2764212

[B3] Krenner HJ Pryor CE He J Petroff PM A semiconductor exciton memory cell based on a single quantum nanostructure Nano Lett 2008 8 6 1750 1755 10.1021/nl800911n 18500845

[B4] Usman M Inoue T Harda Y Klimeck G Kita T Experimental and atomistic theoretical study of degree of polarization from multilayer InAs/GaAs quantum dot stacks Phys Rev B 2011 84 115321

[B5] Čechavičius B Kavaliauskas J Krivaitė G Seliuta D Valušis G Steer MJ Harrison P Halsal MP Photorefectance and surface photovoltage spectroscopy of beryllium-doped GaAs/AlAs multiple quantum wells J Appl Phys 2005 98 023508 10.1063/1.1978970

[B6] Nedzinskas R Čechavičius B Kavaliauskas J Karpus V Seliuta D Tamošiūnas V Valušis G Fasching G Unterrainer K Strasser G Modulated reflectance study of InAs quantum dot stacks embedded in GaAs/AlAs superlattice J Appl Phys 2009 106 064308 10.1063/1.3212980

[B7] Nedzinskas R Čechavičius B Karpus V Kavaliauskas J Valušis G Li LH Khanna SP Linfield EH Photoreflectance and photoluminescence studies of epitaxial InGaAs quantum rods grown with As2 and As4 sources J Appl Phys 2011 109 123526 10.1063/1.3599888 PMC3541080 23127157

[B8] Nedzinskas R Čechavičius B Česnauskas A Kavaliauskas J Karpus V Valušis G Li LH Khanna SP Linfield EH Electronic structure and optical anisotropy of InGaAs quantum rods studied by photoreflectance and photoluminescence Phys Status Solidi C 2012 9 7 1640 1642 10.1002/pssc.201100572

[B9] Ridha P Li LH Mexis M Smowton PM Andrzejewski J Sȩk G Misiewicz J O’Reilly EP Patriarche G Fiore A Polarization properties of columnar quantum dots: effects of aspect ratio and compositional contrast IEEE J Quantum Electron 2010 46 2 197

[B10] Li LH Ridha P Patriarche G Chauvin N Fiore A Shape-engineered epitaxial InGaAs quantum rods for laser applications Appl Phys Lett 2008 92 121102 10.1063/1.2903098

[B11] Misiewicz J Sitarek P Sȩk G Kudrawiec R Semiconductor heterostructures and device structures investigated by photoreflectance spectroscopy Mater Sci (Poland) 2003 21 263

[B12] Aspnes DE Third-derivative modulation spectroscopy with low-field electroreflectance Surf Sci 1973 37 418

[B13] nextnano³ Next generation 3D nanodevice simulator http://www.nextnano.de

[B14] Jang YD Lee H Lee D Kim JS Leem JY Noh SK The energy level spacing from InAs/GaAs quantum dots: its relation to the emission wavelength, carrier lifetime, and zero dimensionality J Appl Phys 2006 99 096101 10.1063/1.2192146

[B15] Shields AJ Klipstein PC Line-shape model for the modulated reflectance of multiple quantum wells Phys Rev B 1991 43 9118 9125 10.1103/PhysRevB.43.9118 9996581

[B16] Glembocki OJ Shanabrook BV Semiconductors and Semimetals 1992 New York: Academic 221

[B17] Li LH Patriarche G Chauvin N Ridha P Rossetti M Andrzejewski J Sȩk G Misiewicz J Fiore A Controlling the aspect ratio of quantum dots: from columnar dots to quantum rods IEEE J Sel Top Quantum Electron 2008 14 4 1204 1213

[B18] Ren HW Sugisaki M Sugou S Nishi K Gomyo A Masumoto Y Lateral composition modulation induced optical anisotropy in InP/GaInP quantum dot system Jpn J Appl Phys 1999 38 2438 2441 10.1143/JJAP.38.2438

[B19] Mukai K Watanabe K Polarization symmetry of vertical photoluminescence from columnar InAs/GaAs quantum dots e-J Surf Sci Nanotech 2009 7 537 540

[B20] Mukai K Watanabe K Kimura Y Grazing incidence X-ray diffraction measurements of columnar InAs/GaAs quantum dot structures Jap J Appl Phys 2010 49 04DH07 10.1143/JJAP.49.04DH07

